# Reliability of left ventricular noncompaction imaging criteria - the fractal facts

**DOI:** 10.1186/1532-429X-15-S1-P136

**Published:** 2013-01-30

**Authors:** Gaby Captur, Andrew Flett, Andrea Barison, Daniel Sado, Anna Herrey, William J McKenna, James Moon

**Affiliations:** 1 Cardiac Imaging Department, The Heart Hospital UK, London, UK; 2 UCL Institute of Cardiovascular Science, University College London, London, UK; 3 Scuola Superiore Sant'Anna, Pisa and Fondazione "G.Monasterio" CNR, Pisa, Italy

## Background

Left ventricular noncompaction (LVNC) is a cardiomyopathy with important prognostic implications. Identification of patients with LVNC using current criteria is challenging. Fractal analysis is a novel approach which summarises global LV trabecular complexity as a continuous variable, the fractal dimension, enabling the establishment of ethnically and disease-appropriate reference ranges for trabeculation.

## Methods

**Figure 1:**
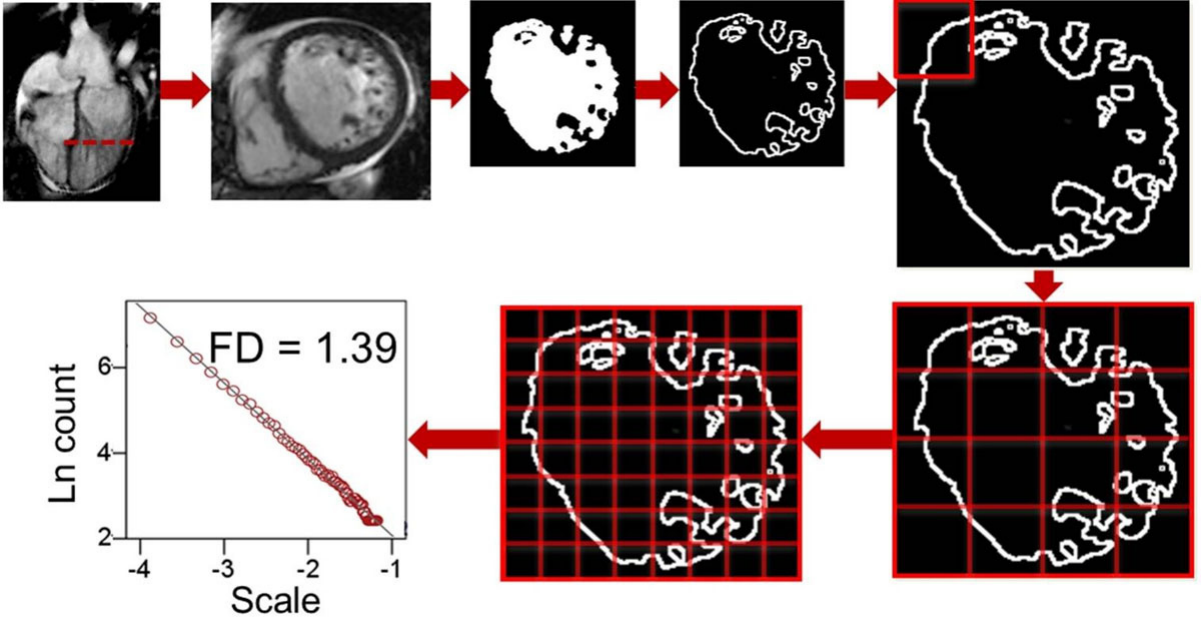
Image processing sequence of one slice out of the LV cine volume stack. Fractal analysis involves dividing 2D space into a grid of boxes and counting the number of boxes that contain part of the skeletonized data. This process is repeated for four different box sizes. The exponent of the line-of-best fit across the points on the log-log plots of box count against scale, represents the FD. LV indicates left ventricle; 2D, two-dimensional; FD, fractal dimension; Ln, logarithm.

We aimed to determine the reproducibility and accuracy of the fractal method compared to other LVNC criteria. We used these results to estimate sample size requirements for population studies of trabeculation. Two independent observers analyzed CMR data from 20 non-selected LVNC cases (mean age 47±13, 11 men) and 40 non-selected healthy volunteers (healthy whites, n=20; healthy blacks, n=20, mean age 45±14, 23 men). Datasets were evaluated using the fractal method (Figure 1) and two other CMR criteria (Petersen and Jacquier). Factorial repeated measures analysis of variance was performed to estimate within-subject variance components and total variability to be used in sample size calculations for the CMR criteria.

## Results

**Figure 2:**
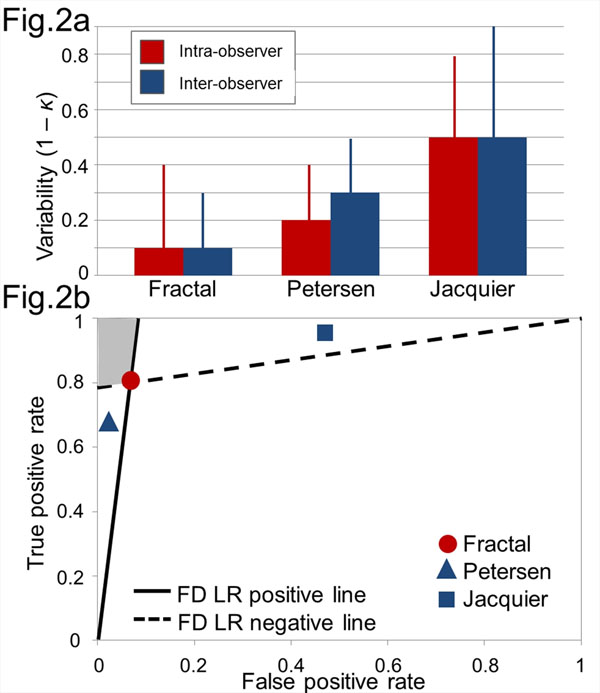
a) Intra and inter-observer variability for the fractal technique and the comparator criteria for LVNC represented as 1 - Fleiss' κ (vertical lines represent 1 - CI). Variability of binary outcome data was greatest for the Jacquier method. Fractal analysis performed most reproducibly across the three populations. b) Likelihood ratios graph showing true positive rate (y axis) plotted against false positive rate (x axis) for the three CMR techniques. The fractal method shows better all-round diagnostic ability compared to the other two tests. LVNC indicates left ventricular noncompaction; κ, kappa; CI, confidence interval; CMR, cardiovascular magnetic resonance; LR, likelihood ratio. Other abbreviations as in Figure 1.

The fractal approach was reproducible (for intra and inter-observer readings: repeatability coefficients, 0.059 and 0.067; intraclass correlation coefficients, 0.98 and 0.97; coefficients of variation, 5%, both). This reproducibility was greater than that calculated for the other LVNC analysis techniques (Figure 2a). Likelihood ratio graphs (Figure 2b) showed superior accuracy for the fractal method over the comparator techniques based on a composite of sensitivity, specificity and positive predictive values. These results translate into superior ability to detect difference and more powerful clinical trials - the minimum number of individuals needed to detect a clinically significant difference in trabeculation between diseased and healthy study populations would be 26 for the fractal method; 60 for the Petersen and 94 for the Jacquier technique (assuming power, 0.90; two-tailed α, 0.05).

## Conclusions

Fractal analysis of LV trabeculae demonstrates good reproducibility and is more accurate in diagnosing LVNC than other techniques. The fractal dimension, if used in clinical trials would more than halve the number of patients required to detect clinically relevant differences in trabecular complexity.

## Funding

J.C.M is supported by the Higher Education Funding Council for England.

G.C. is supported by the University College London through a Graduate Research Scholarship and by the European Union through a Science and Technology Research Grant.

This work was undertaken at the University College London Hospital and University College London, which receive a proportion of funding from the Department of Health's National Institute for Health Research Biomedical Research Centres funding scheme.

